# Grape Seed Proanthocyanidin Extract Ameliorates Diabetic Bladder Dysfunction via the Activation of the Nrf2 Pathway

**DOI:** 10.1371/journal.pone.0126457

**Published:** 2015-05-14

**Authors:** Shouzhen Chen, Yaofeng Zhu, Zhifeng Liu, Zhaoyun Gao, Baoying Li, Dongqing Zhang, Zhaocun Zhang, Xuewen Jiang, Zhengfang Liu, Lingquan Meng, Yue Yang, Benkang Shi

**Affiliations:** 1 Department of Urology, Qilu Hospital of Shandong University, Wenhua Xi Road, Jinan, Shandong Province, People’s Republic of China; 2 Department of Urology, The Central Hospital of Tai’ an, Longtan Road, Tai’ an, Shandong Province, People’s Republic of China; 3 Department of Urology, People’s Hospital of Yinan County, Lishan Road, Yinan, Shandong Province, People’s Republic of China; 4 Department of Geriatrics, Qilu Hospital of Shandong University, Wenhua Xi Road, Jinan, Shandong Province, People’s Republic of China; University of Pécs Medical School, HUNGARY

## Abstract

Diabetes Mellitus (DM)-induced bladder dysfunction is predominantly due to the long-term oxidative stress caused by hyperglycemia. Grape seed proanthocyanidin extract (GSPE) has been reported to possess a broad spectrum of pharmacological and therapeutic properties against oxidative stress. However, its protective effects against diabetic bladder dysfunction have not been clarified. This study focuses on the effects of GSPE on bladder dysfunction in diabetic rats induced by streptozotocin. After 8 weeks of GSPE administration, the bladder function of the diabetic rats was improved significantly, as indicated by both urodynamics analysis and histopathological manifestation. Moreover, the disordered activities of antioxidant enzymes (SOD and GSH-Px) and abnormal oxidative stress levels were partly reversed by treatment with GSPE. Furthermore, the level of apoptosis in the bladder caused by DM was decreased following the administration of GSPE according to the Terminal Deoxynucleotidyl Transferase (TdT)-mediated dUTP Nick-End Labeling (TUNEL) assay. Additionally, GSPE affected the expression of apoptosis-related proteins such as Bax, Bcl-2 and cleaved caspase-3. Furthermore, GSPE showed neuroprotective effects on the bladder of diabetic rats, as shown by the increased expression of nerve growth factor (NGF) and decreased expression of the precursor of nerve growth factor (proNGF). GSPE also activated nuclear erythroid2-related factor2 (Nrf2), which is a key antioxidative transcription factor, with the concomitant elevation of downstream hemeoxygenase-1 (HO-1). These findings suggested that GSPE could ameliorate diabetic bladder dysfunction and decrease the apoptosis of the bladder in diabetic rats, a finding that may be associated with its antioxidant activity and ability to activate the Nrf2 defense pathway.

## Introduction

Grape seed proanthocyanidin extract (GSPE) is chemically composed of a mixture of pycnogenol and flavonoid including oligomeric proanthocyanidins[1], which are potent antioxidants extracted from grape seeds and skins. GSPE has been reported to demonstrate a remarkable spectrum of biological, pharmacological and therapeutic properties against oxidative stress[2, 3]. The antioxidative activities of GSPE were found to be much stronger than those of vitamins C and E[4]. Previous studies have indicated that GSPE showed a protective effect on cardiovascular disease[5], nephropathy[6, 7], atherosclerosis[8], and neuropathy[2, 9], among other conditions. Despite these pharmacological benefits, whether GSPE exerts protective effects on diabetic bladder function and the underlying mechanism remain obscure.

Diabetic bladder dysfunction (DBD), a common complication of DM[10], manifests with an overactive bladder (OAB), urgency, urinary retention, dysuresia and other phenotypes such as decreased sensation, which severely affects the quality of life[11]. The mechanism of DBD is multifactorial, and both the changes in detrusor smooth muscle cells and innervation or function of the neuronal component have been implicated in diabetic cystopathy[12]. Although the etiology and pathogenesis of DBD are complicated, a growing number of studies has shown that oxidative stress plays a significant role in the development of DBD[13]. Some studies have indicated that the apoptosis of smooth muscle cells caused by oxidative stress possibly contributes to DBD[14, 15]. In addition, our previous results demonstrated that the impaired neuronal function and decreased expression of nerve growth factor (NGF) might be another potential mechanism of DBD[16, 17].

Oxidative stress plays crucial role in the injury of both smooth muscle and innervation in the bladder, a phenomenon that leads to DBD. Nuclear erythroid related factor2 (Nrf2) is a transcription factor involved in regulating the cellular antioxidative responses and redox status by promoting the expression of antioxidative genes (phase II genes) through the antioxidant response element (ARE)[18, 19]. The Nrf2 signaling pathway can be activated by many types of phytochemicals as well as food polyphenols[20, 21]. The activation of the Nrf2 signaling pathway that leads to the up-regulation of antioxidative genes protects neural functions and demonstrates large effects on neurodegenerative diseases[20, 22]. In addition, some studies have suggested that the activation of the Nrf2 pathway plays roles in myocyte differentiation as well as muscular contractile and metabolic properties in a diabetic model of muscle atrophy[23]. In some other studies, the activation of the Nrf2 pathway could protect various cells against apoptosis [24].

In the present study, we used the streptozotocin (STZ)-induced type 1 diabetic mouse model to explore whether GSPE could improve diabetic bladder dysfunction. In addition, whether the protective effect of GSPE was associated with the activation of the Nrf2 signal pathway was also investigated.

## Materials and Methods

### Materials and animals

GSPE (comprising 56% dimeric proanthocyanidins, 12% trimeric proanthocyanidins, 6.6% tetrameric proanthocyanidins and small amounts of monomeric and highmolecular-weight oligomeric proanthocyanidins and flavanols) was provided by Jianfeng Inc. (Tianjin, China). The components of GSPE were analyzed using high-performance liquid chromatography with gas chromatography-mass spectrometry detection. Rabbit anti-Nrf2 antibody, mouse anti-HO-1 antibody, rabbit anti-NGF antibody, rabbit anti-histone H2A.X antibody, and rabbit anti-Bax antibody were obtained from Abcam (Cambridge, UK); rabbit anti-bcl-2 antibody, mouse anti-actin antibody, goat-anti-rabbit IgG-HRP, and goat-anti-mouse IgG-HRP were obtained from Santa Cruz Biotechnology(Santa Cruz, CA, USA); rabbit anti-Cleaved-Caspase-3 was obtained from Cell Signaling Technology (Beverly, MA, USA); rabbit anti-pro-NGF was obtained from Sigma Chemical Co.(St. Louis, MO,USA). The total Superoxide Dismutase (SOD) assay kit, Glutathione Peroxidase (GSH-Px) assay kit, and Malondialdehyde (MDA) assay kit were obtained from Nanjing Jiancheng Bioengineering Institute (Nanjing, China). The BCA assay kit, Nuclear and Cytoplasmic Protein Extraction Kit, and TdT-mediated dUTP Nick-End Labeling (TUNEL) Apoptosis Assay Kit were from Beyotime Institute of Biotechnology (Beijing, China).

Wistar rats (Female, 8 weeks old, weighing approximately 250 g) were purchased from the Animal Center of Shandong University (license number: SCXX20090011). The female rats were suitable for the cystometrography. The rats were fed under a 12 h light/dark cycle and had free access to standard rat chow and tap water under the condition of 24°C and 60% humidity. Animal care and management were approved by the Ethics Committee of Qilu Hospital of Shandong University.

### Induction of DM

The rats were fasted for 18 h ahead of the induction of diabetes. Intraperitoneal injection of streptozotocin (50mg/kg body weight; Sigma-Aldrich), which was freshly dissolved in citrate buffer (0.1 M, pH 4.5), was used to induce DM. The control rats were injected with citrate buffer equally. At 72 h after STZ injection, tail venous blood samples were used for the measurement of blood glucose via a glucose strip test in a glucometer (Roche Diagnostics Corporation, Indianapolis, IN, USA). Rats with fasting serum glucose levels above 300 mg/dl were included in the diabetic animal group.

### Experimental design

After adaptation to the laboratory environment, thirty rats were randomly divided into three groups (n = 10, each) as follows: group I, the control group (Control); group II, the untreated diabetic group (DM); group III, the GSPE-treated group (DM/GSPE). The GSPE-treated group was given 250 mg/kg of GSPE, which was in powder form and dissolved in normal saline solution, by oral gavage once daily for 8 weeks. The rats in groups I and II were treated using isometric normal saline. After 8 weeks of feeding, the intravesical pressure was measured, and then the bladder tissue was removed for subsequent analysis.

### Cystometrography

The procedure for recording the intravesical pressure under isovolumetric conditions has been applied in our previous study[25]. Briefly, under successful anesthesia by urethane (1.2 g/kg subcutaneously), a PE-240 catheter was used for tracheal intubation. The bladder was exposed by lower-midline abdominal incision, and then the ureters were cut and tied. A PE-50 catheter was inserted through the bladder dome and was connected with a pressure transducer (Laborie Medical Technologies Inc., Beijing, China) via a three-way stopcock for recording the intravesical pressure and a micromedicine infusion-pump (Hangzhou Zeda Instruments Co., Ltd., Hangzhou, China) for infusing saline into the bladder. Thereafter, saline was slowly pumped into the bladder at 0.05 ml per minute until rhythmic bladder contractions appeared. Finally, the intravesical pressure was recorded in half an hour.

### Bladder tissue preparation

Following cystometrography, rats were sacrificed using the cervical dislocation method. Bladder tissues were removed and divided into four parts immediately. The first part of the bladder tissues in each group was used for paraffin sections. After fixation in 10% formalin solution, the bladder tissues were dehydrated by 70% and 100% alcohol solutions and then embedded in paraffin. Next, 4-μm-thick sections were cut using a microtome and placed on glass slides. Thereafter, hematoxylin and eosin (HE) staining, immunohistochemistry and Terminal Deoxynucleotidyl Transferase (TdT)-mediated dUTP Nick-End Labeling (TUNEL) staining were performed utilizing these sections.

The second part of the tissue was homogenized in ice-cold PBS for the estimation of oxidative stress in the bladder. The third part was homogenized in ice-cold RIPA buffer (Beyotime Institute of Biotechnology) containing 1 mM PMSF (Beyotime Institute of Biotechnology) to extract the total protein of the tissue. The last portion was used for nucleoprotein extraction using a nuclear extraction kit according to the manufacturer’s instructions. The protein concentrations were detected using a BCA assay kit. The prepared tissue homogenate and extracted proteins were stored separately at -80°C for further analyses.

### Bladder histopathological examination and immunohistochemical staining

After regular deparaffinization, the sections were stained with hematoxylin and eosin (HE) and observed from ×100 to ×400 magnifications.

Regular immunohistochemical staining was conducted as previously described[17]. Briefly, SP-9000 SP link Detection Kit (ZSGB-BIO, Beijing, China) and DAB detection kit (ZSGB-BIO) were used for the immunohistochemical studies according to the manufacturer's instructions. Rabbit anti-rabbit Nrf2 antibody was utilized at a dilution of 1/200. Two pathologists conducted the histopathological observations in a blinded manner.

### Estimation of oxidative stress in the bladder

Oxidative stress in the bladder was measured as described previously[17]. GSH-Px, T-SOD, and MDA assays in bladder tissues were estimated by spectrophotometry according to the manufacturer’s protocols. Briefly, GSH-Px activity was measured using the enzyme-catalyzed reaction product (reduced glutathione) and the absorbance was recorded at 412 nm. The measurement of T-SOD activity was based on the combination of xanthine and xanthine oxidase, and the absorbance was read at 550 nm. The MDA level was detected using the thiobarbituric acid (TAB) method and the maximum absorbance was at 532 nm. The activities of GSH-Px and T-SOD are expressed as U/mg pro, while the level of MDA is expressed as nmol/mg pro.

### (TUNEL) Assay to detect the level of apoptosis

The one-step TUNEL apoptosis assay kit was used to detect the apoptosis level in the bladder according to the manufacturer’s instructions. Briefly, the sections were regularly hydrated and incubated with a 3% hydrogen peroxidase solution to remove the endogenous peroxidase. Next, the sections were incubated with the TUNEL mixture in a humidified and dark chamber for 30 minutes. Finally, the sections were stained with DAPI (Sigma-Aldrich) for 10 minute at room temperature to stain cell nuclei. The stained sections were observed under a fluorescence microscope. The apoptotic cells showed red fluorescence excited by light with a wavelength of 550 nm, while the cell nuclei demonstrated blue fluorescence excited by light with a wavelength of 358 nm. The percentage of TUNEL-positive cells was recorded.

### Western blot analysis

Western blot analysis was performed as described previously[25]. Protein (60 ug) was separated by denaturing SDS—PAGE and transferred onto a PVDF Membrane (Millipore, Bedford, MA, USA), utilizing electrophoretic transfer (Bio-Rad, Hercules, CA, USA). The membrane was blocked with 5% non-fat milk for 1 h at room temperature and incubated overnight at 4°C with the primary antibody (in TBST with 5% BSA). Next, the membrane was washed with TBST three times and then incubated with secondary antibodies conjugated with HRP for 2 h. Finally, the bands were revealed using the super ECL plus detection reagent (Applygen Technologies Inc., Beijing, China). Relative protein quantification was measured using Quantity One software (Bio-Rad, Hercules, CA, USA).

### Statistical analysis

All of the data were presented as the mean ± standard error of mean (SEM) (n = 5 at least per group). Comparisons were performed using one-way ANOVA for the different groups followed by Dunnett's test for comparisons between two groups. A P value less than 0.05 was considered to indicate statistical significance. All of the data were analyzed using the Statistical Package for Social Sciences (Version19.0; SPSS, Chicago, IL, USA).

## Results

### Effects of GSPE on general characteristics of diabetic rats

As shown in Table 1, diabetic rats displayed a statistical weight loss despite taking in more food and water than control rats (241.41±7.38 g versus 311.92±4.69 g, respectively; P = 0.000) at the end of 8 weeks. GSPE obviously increased the body weight of the diabetic rats (272.86±6.89 g versus 241.41±7.38 g, respectively; P = 0.038). However, GSPE had no obvious effects on the blood glucose level at 8 weeks (444.27±8.19 mg/dl versus 455.39±8.30 mg/dl, respectively; P = 0.717). In addition, the urine volume per day was significantly increased in diabetic rats than in control animals (65.32±1.74 ml versus 16.95±0.89 ml, respectively; P = 0.000), a situation that aggravated the burden of the bladder. Nevertheless, GSPE did not influence the urine volume of the diabetic rats (60.53±2.92 ml versus 65.32±1.74 ml, respectively; P = 0.433).

**Table 1:** General characteristics of the three groups.

General characteristics	Control	DM	DM+GSPE
**Body weight at 0 weeks (g)**	242.42±1.40	242.61±1.36	242.84±1.54
**Body weight at 8 weeks (g)**	311.92±4.69	241.41±7.38^a^	272.86±6.89^b^
**Blood glucose at 8 weeks (mg/dl)**	95.17±3.31	455.39±8.30^c^	444.27±8.19
**Urine volume per day (ml)**	16.95±0.89	65.32±1.74 ^d^	60.53±2.92

The values represent the mean ± SEM of 10 animals per group;

^a^, n = 10, P = 0.000 vs. Control

^b^, n = 10, P = 0.038 vs. DM

^c^, n = 10, P = 0.000 vs. Control

^d^, n = 10, P = 0.000 vs. Control

### GSPE protected against diabetic bladder dysfunction

**Fig 1:**
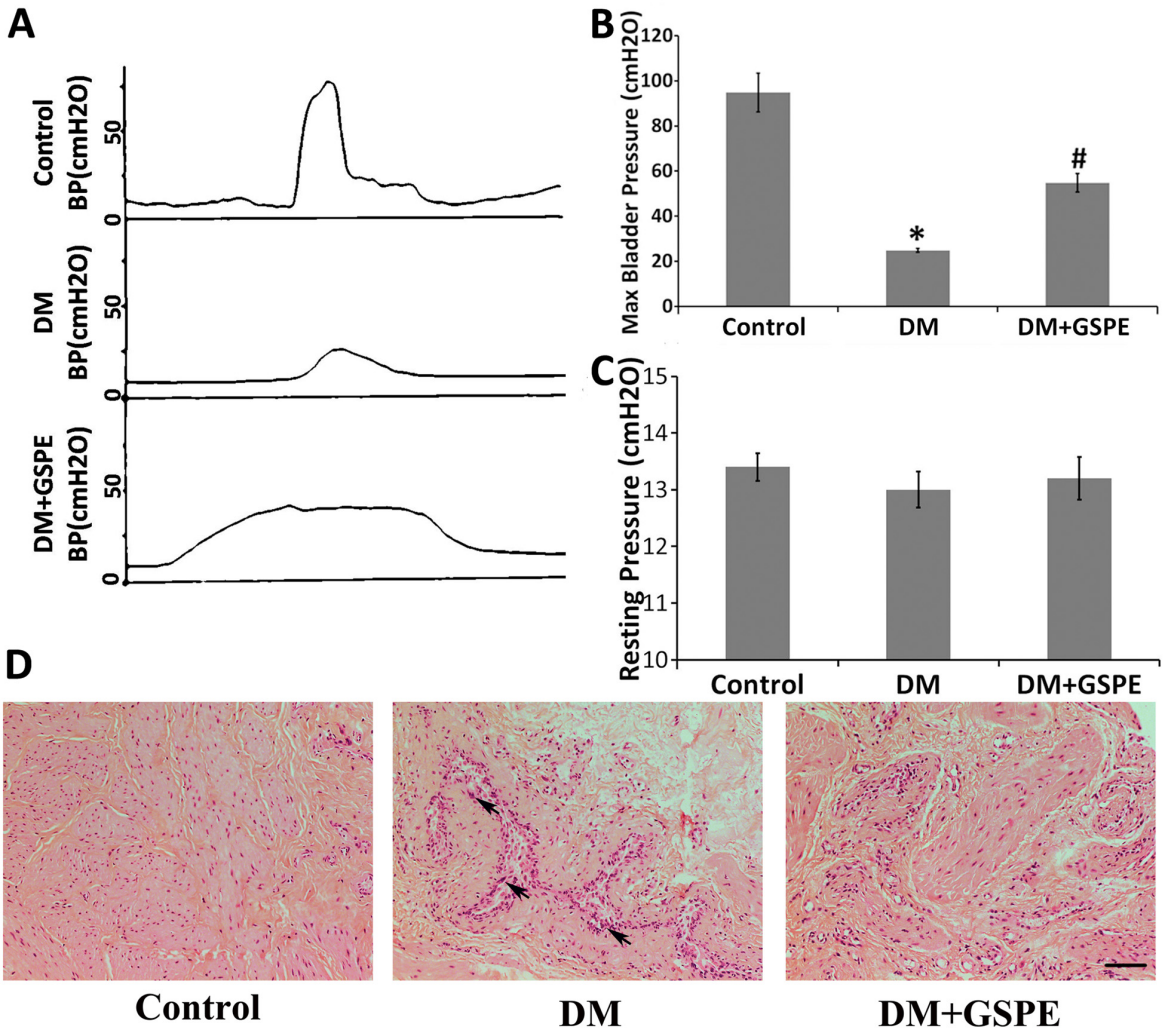
Functional and morphological evaluation of the bladder in the control, DM, DM/GSPE group. A, cystometrograms of the three groups; B, the statistical results of maximal bladder pressure. * n = 5, P = 0.003 vs. the Control group. # n = 5, P = 0.004 vs. the DM group; C, the statistical results of resting pressure; D, histological sections of the bladder via HE staining showed that there was structural damage and inflammatory cell infiltration in the DM group while GSPE could improve the morphological changes. The arrow shows inflammatory cells infiltration. Scale bar, 20 μm.

Cystometrography was performed to show the change in bladder function in different groups as previously described [15, 26]. We mainly focused on the change in the maximal detrusor pressure and resting pressure, which represented the contractile function of the bladder. We found that the maximal detrusor pressure was significantly decreased in diabetic rats compared to control animals (P = 0.003) (Fig 1A and 1B). Application of GSPE had a statistical influence on maximal detrusor pressure compared to the DM group (P = 0.004) (Fig 1A and 1B). However, there were no significant changes about the resting pressure in different groups (Fig 1A).

### GSPE improved DM induced histological changes in bladder

As displayed in Fig 1C, the results of HE staining showed that GSPE possessed the protective effect on diabetic bladder. At the end of 8 weeks, the bladder tissues in DM group were thinner than control group. DM caused obvious histological changes including structure damage of the detrusor smooth muscle and inflammatory cells infiltration in rat bladder tissues. Treatment with GSPE significantly alleviated the histological damage in bladder of diabetic rats (Fig 1C).

### GSPE attenuated the oxidative stress in diabetic bladder

**Fig 2:**
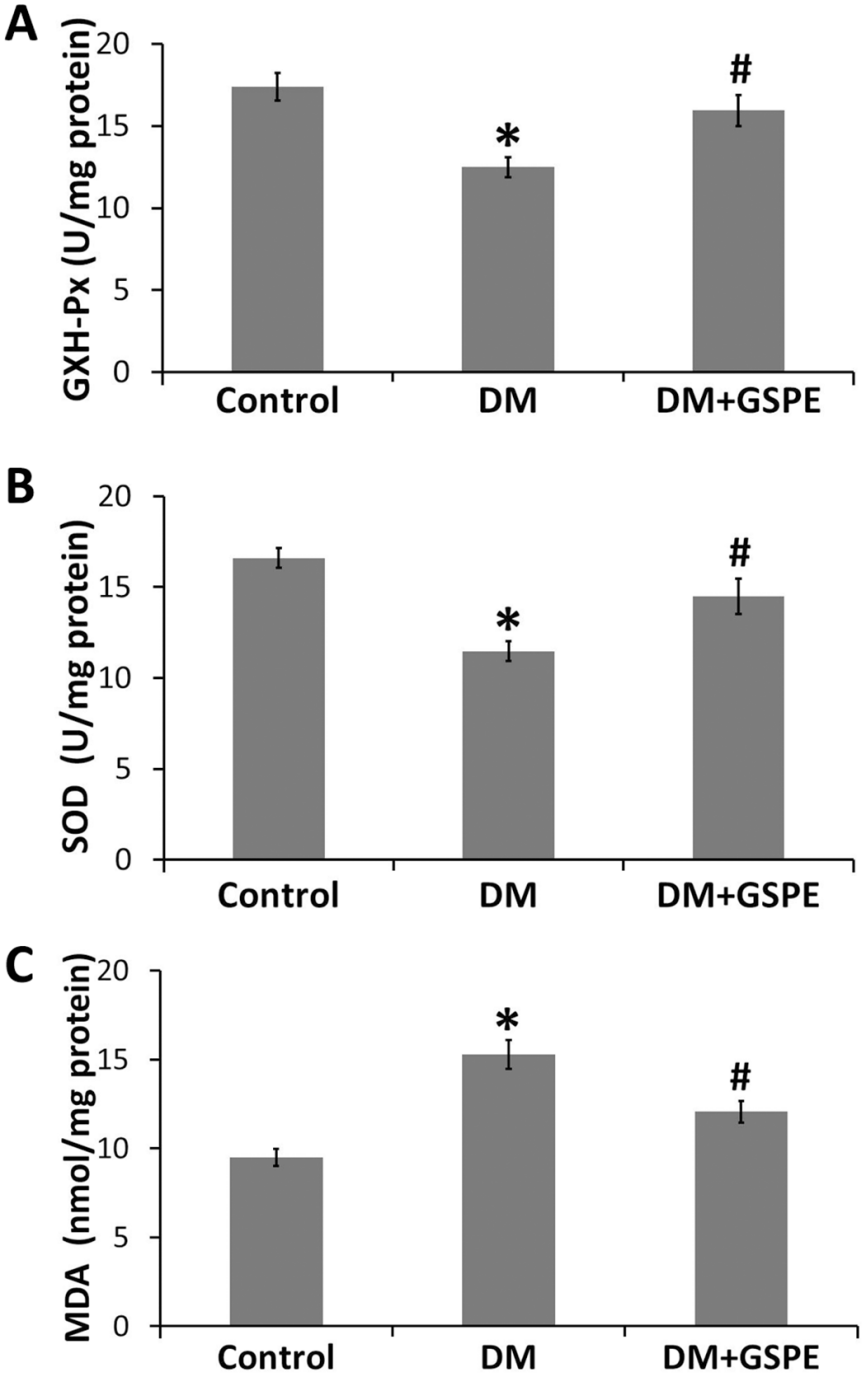
Effect of GSPE on oxidative stress in the bladder. A, GXH-Px activity in the bladder of the three groups, * n = 9, P = 0.001 vs. the Control group. # n = 9, P = 0.025 vs. the DM group; B, T-SOD activity in the bladder of the three groups, * n = 9, P = 0.000 vs. the Control group. # n = 9, P = 0.023 vs. the DM group; C, MDA level in the bladder of the three groups, * n = 9, P = 0.000 vs. the Control group. # n = 9, P = 0.013 vs. the DM group.

Many studies demonstrated that the activity of SOD, GXH-Px and the level of MDA was the indicators of oxidative stress status[17, 27]. Our studies showed that the activities of GXH-Px and SOD in bladder were significantly decreased in diabetic rats by 28% and 34%, respectively, compared with the controls (P<0.01). However, treatment with GSPE could restore the GXH-Px and T-SOD activities significantly (P = 0.025 and P = 0.023, respectively) (Fig 2A and 2B). The level of MDA in the bladder compared with the controls was elevated markedly due to the influence of DM (P = 0.000). However, the results also revealed that the level of MDA was decreased significantly via the administration of GSPE (P = 0.013) (Fig 2C).

### Protective effects of GSPE on apoptosis in the diabetic bladder

**Fig 3:**
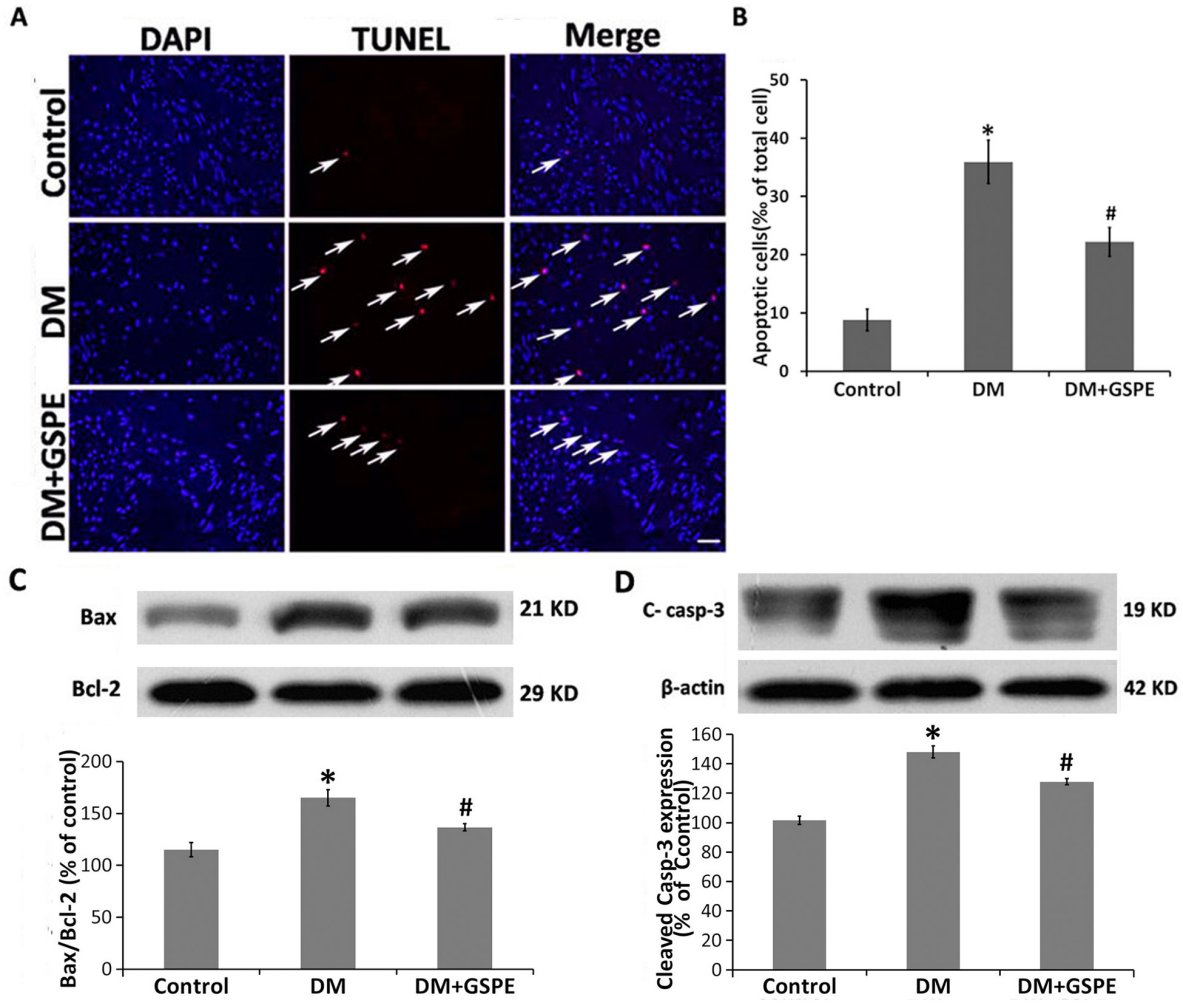
Effect of GSPE on apoptosis in the bladder. A. TUNEL staining showing the cell apoptosis of the bladder in the control, DM and DM/GSPE groups. A nucleus stained with DAPI (blue); the arrow shows TUNEL staining (red). Scale bar, 50 μm. B. The statistical results of TUNEL staining, * n = 5, P = 0.002 vs. the Control group. # n = 5, P = 0.049 vs. the DM group. C. The protein expression of Bax to Bcl2 ratio in the bladder of the three groups, * n = 7, P = 0.001 vs. the Control group. # n = 7, P = 0.028 vs. the DM group. D. The protein expression of cleaved caspase-3 in the bladder of the three groups, * n = 5, P = 0.000 vs. the Control group. # n = 5, P = 0.036 vs. the DM group.

Because the diabetic bladder dysfunction was associated with the apoptosis level of the bladder caused by oxidative stress[14, 28], we next examined whether GSPE had any protective effects on the apoptosis level of the diabetic bladder by TUNEL staining. The number of TUNEL-positive cells in the bladder of diabetic rats was markedly increased compared with the control animals (P = 0.002), whereas GSPE statistically decreased the bladder TUNEL-positive cells of diabetic rats (P = 0.049) (Fig 3A and 3B). In accordance with TUNEL results, Western blotting demonstrated a significant increase in the expression of cleaved-caspase-3 in the bladder of DM rats (P = 0.000), while treatment with GSPE could significantly decrease the expression of cleaved-caspase-3 (Fig 3D) (P = 0.036).

Because previous studies have indicated that diabetic cystopathy was closely related to mitochondrial apoptotic pathway-mediated bladder apoptosis [28], we next investigated whether GSPE could avert the activation of the mitochondrial apoptotic pathway in the diabetic bladder. The Bax to Bcl2 expression ratio was significantly increased in the bladder of DM rats compared with the control animals according to Western blot analysis (P = 0.001). GSPE significantly averted the increase in the Bax to Bcl2 expression ratio in the bladder of DM rats (P = 0.028) (Fig 3C).

### GSPE restored the balance of the expression of NGF and proNGF in the diabetic bladder

**Fig 4:**
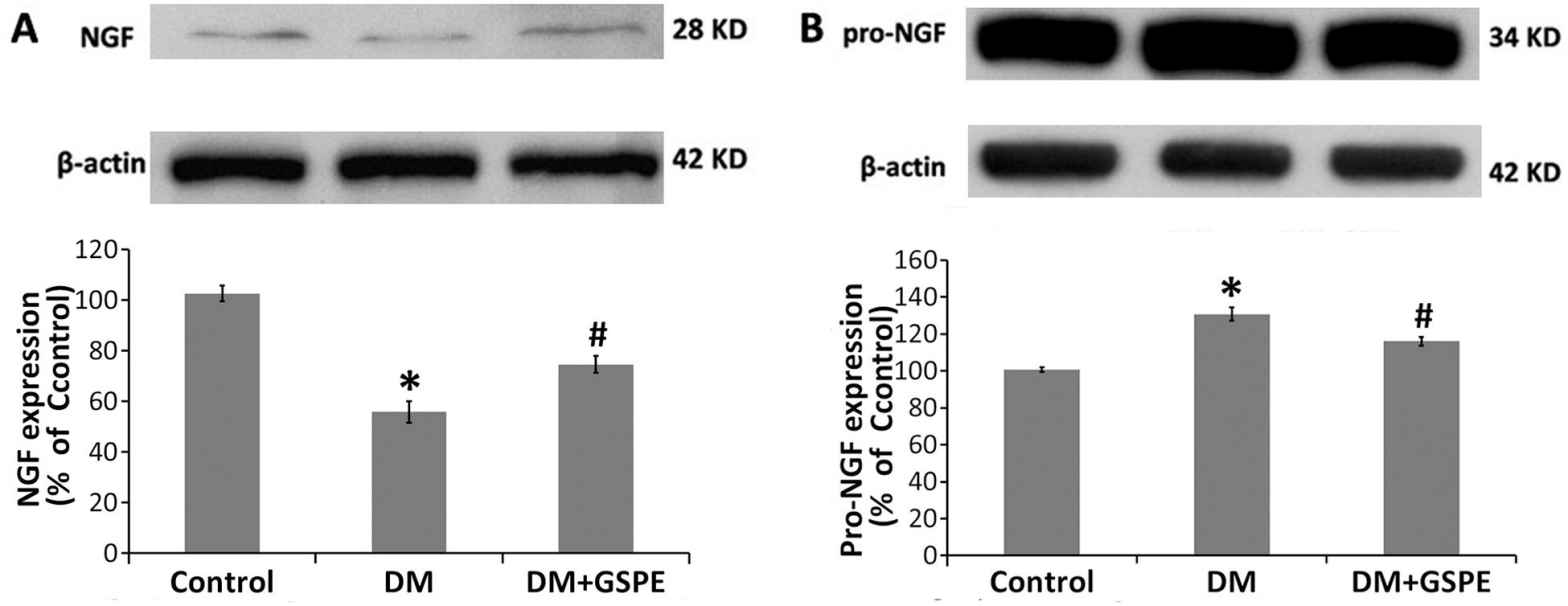
Effect of GSPE on the expression of NGF and proNGF in the bladder. A. The protein expression of NGF in the bladder of the three groups, * n = 5, P = 0.000 vs. the Control group. # n = 5, P = 0.025 vs. the DM group. B. The protein expression of proNGF in the bladder of the three groups, * n = 5, P = 0.001vs. the Control group. # n = 5, P = 0.031vs. the DM group.

DBD was partly caused by DM-induced peripheral neuropathy with the reduced expression of NGF[29]. Because the changes in the levels of NGF and proNGF were associated with DM-induced neural injury[30], we next examined the expression of NGF and proNGF in the bladder of different groups. As shown in Fig 4, the expression of NGF was significantly decreased in the DM group compared with the controls (P = 0.000), while the expression of proNGF was increased (P = 0.001). After the administration of GSPE, the balance of proNGF/NGF was restored. The expression of NGF was increased in the DM/GSPE group compared with the DM group (P = 0.025), while the expression of proNGF was statistically reduced (P = 0.031).

### The protective effects of GSPE involved the activation of Nrf2 pathway

**Fig 5:**
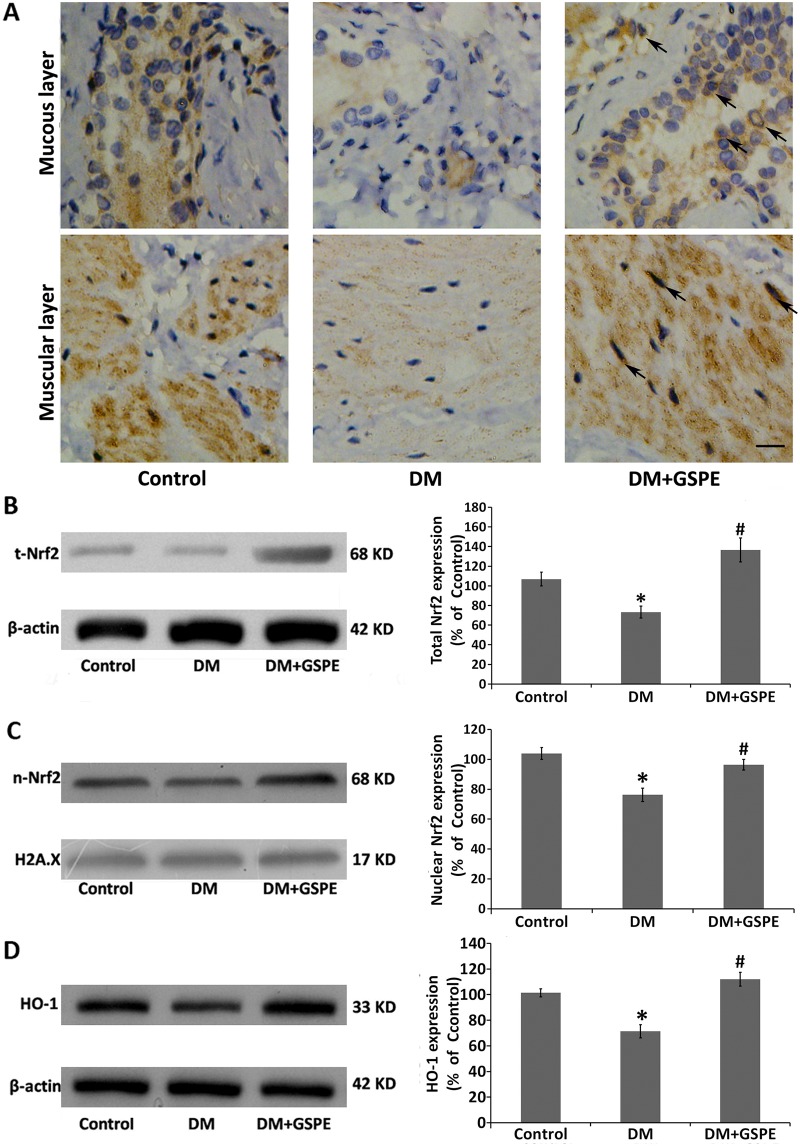
Effect of GSPE on the Nrf2 pathway in the bladder. A. IHC staining results of Nrf2 in the mucous and muscular layers of bladder tissues. The arrow shows Nrf2 positive staining is mainly located in the nucleus of the bladder cells. Scale bar, 50 μm. B. The protein expression of total Nrf2 in the bladder of the three groups, * n = 7, P = 0.011 vs. the Control group. # n = 7, P = 0.004 vs. the DM group. C. The protein expression of nuclear Nrf2 in the bladder of the three groups, * n = 7, P = 0.003 vs. the Control group. # n = 7, P = 0.018 vs. the DM group. D. The protein expression of HO-1 in the bladder of the three groups, * n = 5, P = 0.005 vs. the Control group. # n = 5, P = 0.002 vs. the DM group.

The transcription factor Nrf2 is a vital mediator involved in regulating cellular antioxidative responses. Activation of the Nrf2 pathway leads to the inhibition of apoptosis[24] and neuroprotective effects[20, 22]. We next investigated the effects of GSPE on the expression of Nrf2. As shown in Fig 5A, immunohistochemical results demonstrated that the expression of Nrf2 was qualitatively increased both in the mucous and muscular layers of the bladder by the treatment with GSPE compared with the DM group. However, the expression of Nrf2 was declined in the bladder of the DM group compared with the controls. More importantly, the expression of Nrf2 was mainly located in the nucleus of the bladder cells. Western blotting also showed that the expression of Nrf2 was significantly increased in the bladder of the DM/GSPE group compared with the DM group (P = 0.004), while Nrf2 was decreased in the bladder of the DM group compared with the controls (P = 0.011) (Fig 5B).

Previous studies have shown that Nrf2, as an antioxidative transcription factor, plays a role only by entering the nucleus[21]; thus, we measured the expression of Nrf2 in the nucleus. Consistent with previous studies, the level of Nrf2 in the nucleus was also markedly increased in the DM/GSPE group compared with the DM group (P = 0.018), and the level of Nrf2 in the nucleus was significantly decreased in the DM group compared with the controls (P = 0.003) (Fig 5C).

We next investigated the antioxidative function of Nrf2 by measuring the expression of its downstream target gene HO-1. As shown in Fig 4D, the expression of HO-1 was increased in the DM/GSPE group compared with the DM group (P = 0.002), a finding that was consistent with the expression of Nrf2 in the nucleus. However, our results showed that no significant difference in the expression of total Nrf2, nuclear Nrf2 and total HO-1 could be observed in the bladder from the DM/GSPE group compared with that from the controls.

## Discussion

The number of diabetic patients is increasing greatly, and diabetics are subjected to various costly complications. Diabetic bladder dysfunction (DBD), which is also called diabetic cystopathy[31], is one of the most common complications of DM[11, 32]. The clinical DBD manifestations consist of storage and voiding problems, which substantially affect the quality of life[33, 34]. Previous studies have shown that the pathogenesis of DBD was associated with the myogenic and neurogenic alterations of the bladder[11, 16, 35, 36]. In our studies, we demonstrated that the contractile function of the bladder was damaged significantly in diabetic groups along with the morphological changes (Fig 1). However, we proved here for the first time that the administration of GSPE for 8 weeks could significantly increase the pressure of bladder detrusor and ameliorate the histopathologic changes of the bladder (Fig 1). These results indicated that GSPE could protect rats against DM-induced dysfunction and morphological damage of the bladder.

Previous studies have shown that oxidative stress caused by hyperglycemia plays important roles in the pathogenesis of DBD[11–15]. For instance, the decrease in detrusor smooth muscle force induced by DM was related to the high level of lipid peroxidation products, high expression of aldose reductase and activation of the polyol pathway, all of which were ascribed to oxidative stress[37]. Oxidative stress could also induce apoptosis of the detrusor smooth muscle, which could lead to caused DBD[14, 15, 28]. Oxidative damage might cause neurodegeneration in the bladder by interfering with neurotrophins, such as NGF, for neuron survival[16, 29, 38]. Consistent with previous studies, our results showed that the level of MDA, as an indicator of lipid peroxidation, was higher in the bladder of the DM group than in controls. The activities of GSH-Px and SOD, as markers of redox status, were significantly decreased in the bladder of the DM group than in the controls (Fig 2). GSPE has been reported to possess the prominent properties against oxidative stress[2, 3]. In the present study, GSPE decreased MDA production in the bladder of the DM group (Fig 2C), a finding that may be ascribed to the potent antioxidant activities of GSPE. We also found that GSPE significantly enhanced the activities of GSH-Px and SOD in the bladder of diabetic rats (Fig 2A and 2B). Our results suggested that GSPE could partly alleviate oxidative stress by decreasing lipid peroxide and increasing the activities of antioxidative enzymes in the bladder of diabetic rats.

Cell apoptosis induced by oxidative stress is an important mechanism of diabetic bladder dysfunction[14, 28, 39]. A previous study showed that oxidative stress induced apoptosis in the bladder via the activation of the mitochondrial apoptotic pathway[28]. In accordance with the previous studies, our results showed that the apoptotic level of the bladder was significantly increased in the DM group compared with that in the controls via the TUNEL staining (Fig 3A and 3B). The increased expression of the ratio of Bax to Bcl2 content in the bladder of diabetic rats showed the activation of the mitochondrial apoptotic pathway (Fig 3C). Cleaved caspase-3 was the essential apoptotic initiator and was also activated in the bladder of diabetic rats (Fig 3D). Many studies have shown that GSPE possessed the protective effect on apoptosis induced by oxidative stress[40–43]. Consistently, we found that administration of GSPE could significantly decrease the apoptotic level in the bladder of the DM group by down-regulating the expression of the ratio of Bax to Bcl2 (Fig 3). Thus, our findings suggested that GSPE might protect the bladder against apoptosis induced by oxidative stress via decreasing the Bax/Bcl2 ratio.

As previously described, the changes in the NGF level, which led to neurodegeneration in the bladder, is a possible mechanism of DBD. We next discussed the changes in the expression of NGF in the bladder of diabetic rats. Numerous studies have demonstrated that the balance between the proNGF and NGF levels played important roles in homeostasis. The disruption of the balance possibly led to neurodegeneration, which might further lead to many diseases such as Alzheimer’s disease[30, 44]. ProNGF, as the precursor of NGF, has distinct functions from NGF. Specifically, proNGF with a higher affinity for p75 ^NTR^ determined apoptotic signaling, while NGF with a higher affinity for TrkA^NTR^ activated the trophic signaling pathway[45, 46]. Our previous studies also indicated that the changes in the proNGF/NGF balance might be a possible mechanism of diabetic urethral dysfunction, leading to the changes in the expression of α receptor[25]. In the present study, we found that the balance of the proNGF and NGF level was disrupted in the bladder of diabetic rats. The expression of proNGF was increased significantly in the bladder of DM group compared with that in the controls, while the expression of NGF was decreased (Fig 4). GSPE had a protective effect on peripheral nerves in STZ-induced diabetic rats[9]. Another study showed that the intervention of GSPE was possibly helpful in the treatment of Alzheimer’s disease by decreasing the levels of a memory-impairing Aβ oligomer[47]. In the present study, GSPE could significantly increase the expression of NGF and decrease the level of proNGF in the bladder of DM rats(Fig 4). Thus, in the present study, we demonstrated that GSPE could restore the balance of the proNGF/NGF level in the bladder of diabetic rats for the first time.

The Nrf2 signaling pathway is known to exert antioxidative effects on various cells[18, 19]. Activation of the Nrf2 signaling pathway was reported to protect various cells against apoptosis[24, 48]. Nrf2 was reported to regulate NGF mRNA expression in glioblastoma cells and normal human astrocytes[49]. Some studies have shown that the activation of the Nrf2 pathway could increase the expression of NGF to promote neurite outgrowth in high glucose-treated neurons.[50]. In addition, food polyphenols could activate the Nrf2 signal pathway[20, 21]. In the present study, we first found that the administration of GSPE could enhance the activity of the Nrf2 pathway. After the administration of GSPE, the expression of Nrf2 in the bladder was increased significantly compared with the DM groups (Fig 5A and 5B). Because Nrf2, as a transcription factor, plays a role in the nucleus, we next found that the expression of nuclear Nrf2 was also markedly increased in the bladder of the DM/GSPE group compared with the DM group (Fig 5C). The expression of HO-1, the downstream gene of the Nrf2 pathway, was consistent with the expression of Nrf2 (Fig 5D). In addition, the changes in the activity of SOD and GPH-Px also agreed with the changes in the Nrf2 pathway (Fig 2A and 2B). Thus, our findings suggested that the protective effects of GSPE on the bladder of diabetic rats might be due to the activation of the Nrf2 signaling pathway.

However, there are still a few restrictions in the present study. We used the STZ-induced type 1 DM model to investigate DBD, which was consistent with most previous studies[12, 35], while most of the patients with DBD have type 2 DM clinically. There are some differences between type 1 and type 2 DM such as the insulin level, blood glucose level and metabolic properties[51]. However, the type 1 DM model is widely used to simulate the pathogenesis of DBD because the damage induced by hyperglycemia is the primary cause of DBD. In this study, we did not provide insulin treatment for DM group. It is known that insulin is able to interact with NGF receptor TrkA in PC12 cells[52] and NGF plays an important role in the neurological function of the bladder. So insulin may have influence on the neurological function of the bladder. In addition, in the present study, we did not use the dose gradients in the administration of GSPE, partly because we found that the protective effect of GSPE on diabetic rats was excellent and safe at the dose of 250 mg/kg in our previous studies[53]. Although GSPE is extracted from natural plants and is relatively safe, the side effects and pharmacokinetics of GSPE should be further investigated in the future.

**Fig 6:**
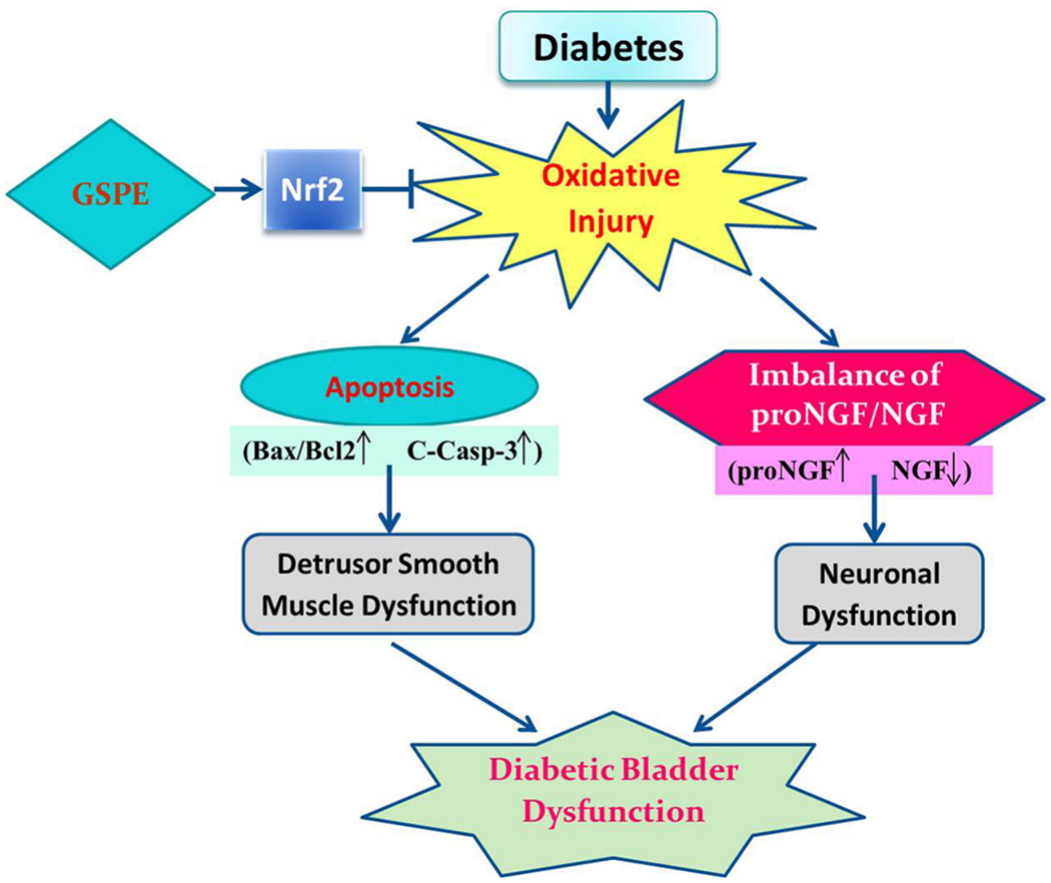
Schematic diagram of the protective mechanism for GSPE in DBD. “→” indicates activation or induction, and “|−” indicates inhibition or blockade. DM induces oxidative stress in the bladder. The enhanced oxidative stress induces apoptosis of the bladder by activating the mitochondrial pathways, leading to the detrusor smooth muscle dysfunction. At the same time, oxidative stress also disrupts the balance of the proNGF and NGF level, leading to neuronal dysfunction of bladder. Detrusor smooth muscle dysfunction and neuronal dysfunction are two important mechanisms of DBD. GSPE treatment attenuates DM-induced oxidative damage most likely by enhancing and activating Nrf2. Additionally, the use of GSPE decreases the level of apoptosis and retains the balance of the proNGF/NGF level. Finally, the use of GSPE improves detrusor smooth muscle dysfunction and neuronal dysfunction. Therefore, the use of GSPE ameliorates diabetic bladder dysfunction.

In summary, our study demonstrated for the first time that GSPE has significant protective effects against diabetic bladder dysfunction, a finding that may be associated with the activation of the Nrf2 pathway in the bladder of diabetic rats. As illustrated in Fig 6, DM-induced oxidative stress triggers apoptosis of the bladder by activating mitochondrial pathways, leading to detrusor smooth muscle dysfunction. At the same time, oxidative stress disrupts the balance of the proNGF and NGF level, leading to neuronal dysfunction of bladder. Detrusor smooth muscle dysfunction and neuronal dysfunction are two important mechanisms of DBD. GSPE treatment attenuates DM induced oxidative damage by enhancing and activating Nrf2. Next, GSPE diminished the apoptotic level of the bladder and restored the balance of the proNGF and NGF levels. Thus, the administration of GSPE ameliorates DM-induced bladder dysfunction and histopathologic changes. Therefore, the administration of GSPE in diabetic patients is promising and, in the future, may be recommended to protect against diabetic bladder dysfunction.
